# The Endowment of Motherhood

**Published:** 1919-07-26

**Authors:** 


### The Endowment of Motherhood.

Endowment of motherhood has been put forward in some of the election addresses of candidates for seats in the first after-war Parliament. It is a vague term not defined by those who use it, but it is certainly understood by many to mean that every woman who bears a child is to be paid some sum by the State for having done so. Various amounts of payment and various periods of time have been suggested. Most people assume that only the wives of men earning less than a certain sum per week shall receive the money. Some would stipulate that only women who have made a contract of marriage should be eligible.

The Unmarried Mother.

Others wish to include "the unmarried mother." As a matter of fact the poor married woman can get a small endowment in the form of a thirty-shilling maternity grant or help from the rates, and the unmarried woman and her offspring are kept, if it be necessary, by money derived from the rates; but neither of these payments is the form of endowment demanded. It is worth while in this period of reconstructional ferment, a time in which there is great danger of rash, ill-considered, though well-meant, legislation passing Parliaments, to consider the proposition and the assumptions on which it is based; for every opinion and shade of opinion rests on assumptions, though assumptions are not usually put into definite shape by the majority of holders of opinions.

The demand for the endowment of motherhood is based on: (1) Every woman has a right to have children; (2) the State requires a great increase of population; (3) every woman renders a service to the State when she bears a child; (4) it is every woman's duty to have children. All four propositions are arguable, and as regards the first most people under Western civilisations do not really recognise the right unless the woman has been able to induce a man to contract a marriage with her. It is very "advanced" to extend the right to all women. The other three are of more practical importance, and one hangs on another, but none is undisputed.

Effect on Birth-Rate.

It is not proposed to examine the *pros* and *cons*, but only to consider some of the probable results of legislation. Is it likely that legislation will lead to any increase in the number of children the more prudent, ambitious, and far-seeing of the population will bear? It is very doubtful, unless the payment for breeding be made so large a sum as to be burdensome taxation. Whether the prudent breed or no the result will be to remove from the imprudent the last vestige of prudential consideration. In fact it will be an excellent trade for a vicious, idle, dissolute man to take to himself a wife and multiply his species through her and to live on her earnings as a mother. The legislation proposed may increase the gross number of citizens, but at no gain in quality. It is probable that those in favour of endowment of motherhood have no desire that the State should interfere with free choice in marriage, but since it is likely such interference will result from such legislation in time, and after mischief has resulted, would it not be better to declare in advance that good breeding is a trade or profession for which women ought to be paid, and then for the State to select in advance of pregnancy the men and women whom it is prepared to pay for producing population, and not leave the matter to promiscuous, unguided chances?

